# COVID-19 Booster Dose Vaccination Coverage and Factors Associated with Booster Vaccination among Adults, United States, March 2022

**DOI:** 10.3201/eid2901.221151

**Published:** 2023-01

**Authors:** Peng-jun Lu, Anup Srivastav, Kushagra Vashist, Carla L. Black, Jennifer L. Kriss, Mei-Chuan Hung, Lu Meng, Tianyi Zhou, David Yankey, Nina B. Masters, Hannah E. Fast, Hilda Razzaghi, James A. Singleton

**Affiliations:** Centers for Disease Control and Prevention, Atlanta, Georgia, USA (P.-j. Lu, A. Srivastav, K. Vashist, C.L. Black, J.L. Kriss, M.-C. Hung, L. Meng, T. Zhou, D. Yankey, N.B. Masters, H.E. Fast, H. Razzaghi, J.A. Singleton);; Leidos Inc., Atlanta (A. Srivastav, M.-C. Hung, T. Zhou);; Oak Ridge Institute for Science and Education, Oak Ridge, Tennessee, USA (K. Vashist)

**Keywords:** COVID-19, coronavirus disease, respiratory infections, booster vaccination, coverage, factors, dose, provider recommendation, vaccination coverage, factors, adults, National Immunization Survey-Adult COVID Module, zoonoses, United States

## Abstract

The Centers for Disease Control and Prevention recommends a COVID-19 vaccine booster dose for all persons >18 years of age. We analyzed data from the National Immunization Survey–Adult COVID Module collected during February 27–March 26, 2022 to assess COVID-19 booster dose vaccination coverage among adults. We used multivariable logistic regression analysis to assess factors associated with vaccination. COVID-19 booster dose coverage among fully vaccinated adults increased from 25.7% in November 2021 to 63.4% in March 2022. Coverage was lower among non-Hispanic Black (52.7%), and Hispanic (55.5%) than non-Hispanic White adults (67.7%). Coverage was 67.4% among essential healthcare personnel, 62.2% among adults who had a disability, and 69.9% among adults who had medical conditions. Booster dose coverage was not optimal, and disparities by race/ethnicity and other factors are apparent in coverage uptake. Tailored strategies are needed to educate the public and reduce disparities in COVID-19 vaccination coverage.

A COVID-19 vaccine booster dose is intended to boost the immune system for better, long-lasting protection when the primary vaccine response decreases over time. Studies have shown that a booster increased the immune response in trial participants who completed a Pfizer-BioNTech (https://www.pfizer.com) or Moderna (https://www.modernatx.com) primary series 6 months earlier or who received a Johnson & Johnson/Janssen (https://www.jnj.com) single-dose vaccine 2 months earlier (*1**,**2*).

With an increased immune response, booster doses provide additional protection against both Delta and Omicron variants for clinical COVID-19 emergency department visits and hospitalization even for those persons who have received an initial vaccine series (*1**,**3*). For example, the mRNA vaccine effectiveness (VE) against emergency room visits during the period of Delta predominance was 76%–86% after the second initial dose and 94% after a booster dose; estimates of VE during Omicron variant predominance were 38%–52% after the second initial dose and 82% after a booster dose. VE against hospitalizations during the period of Delta predominance was 81%–90% after the second initial dose and 94% after a booster dose, and estimates of VE for during Omicron variant predominance were 57%–81% after the second initial dose and 90% after a booster dose (*3*).

The Centers for Diseases Control and Prevention (CDC) first recommended booster doses for select populations in September 2021 and on November 29, 2021, recommended that all persons >18 years of age should get a booster dose when eligible (*1**,**2*). By March 2022, approximately 84% of American adults were fully vaccinated with the COVID-19 primary vaccine series; primary vaccine series completion rates varied by some social‒demographic characteristics (*4*). Receiving a COVID-19 booster dose is useful both to prevent COVID-19-related illness and death and slow the spread of COVID-19 in the United States. The objective of this study was to assess COVID-19 booster dose vaccination coverage by demographics and behaviors and experiences toward vaccination among fully vaccinated adults by using data from the National Immunization Survey–Adult COVID Module (NIS-ACM) (*5*).

## Methods

We collected the NIS-ACM data used in this report by telephone interview among adults >18 years of age by using a random-digit‒dialed sample of cell telephone numbers. 

Data were collected during February 27–March 26, 2022. Trend analysis was based on data collected during October 31, 2021–March 26, 2022. Booster dose was defined as receipt of a third dose of COVID-19 vaccine after completion of a 2-dose primary mRNA COVID-19 vaccine series for adults who are not immunocompromised or a fourth dose of COVID-19 vaccine after completion of a 3-dose mRNA COVID-19 vaccine series for adults who reported being immunocompromised. For respondents whose initial vaccine was a Janssen/Johnson & Johnson vaccine, booster dose was defined as receipt of a second dose of the vaccine after completion of a single-dose primary vaccine series for adults who are not immunocompromised or a third dose of Janssen vaccine after completion of 2-dose series for adults who reported being immunocompromised (*1**,**2*). Receipt of a booster dose of COVID-19 vaccine was based on responses to the questions, “Have you received at least one dose of a COVID-19 vaccine?,” “Which brand of COVID-19 vaccine did you receive for your first dose?,” “How many doses of a COVID-19 vaccine have you received?,” and self-reported health conditions that may put respondents at higher risk for COVID-19 (including immunocompromised status).

Survey questions also collected information on vaccine confidence, behaviors, and experiences, such as being concerned about getting COVID-19, thinking COVID-19 vaccines are safe, believing COVID-19 vaccines are useful for protection from COVID-19, whether friends or family were vaccinated, and whether the respondent had difficulty getting a COVID-19 vaccine (e.g., difficulty getting an appointment online, knowing where to get vaccinated, getting to vaccination sites). Information on demographic characteristics, health insurance status, reported medical conditions, previous diagnosis of COVID-19, disability status, frontline/essential work status, provider recommendation of a COVID-19 vaccine, and work/school COVID-19 vaccination requirement were also collected (*6*). Questions regarding vaccine confidence, behaviors, and experiences did not specifically address booster doses. Analytic datasets were created for approximate months of data collection, and we used data from 5 data collection periods (November 2021, collected during October 31–November 27; December 2021, collected during November 28–December 31; January 2022, collected during January 2–January 29; February 2022, collected during January 30–February 26; and March 2022, collected during February 27–March 26) for these analyses. The response rates for the 5 monthly datasets ranged from 21.4% to 22.0%, and the total sample sizes for the 5 periods were 39,508, 68,612, 62,693, 58,488, and 63,072, respectively.

We stratified COVID-19 booster dose vaccination coverage by using demographic characteristics and vaccine confidence, behaviors, and experiences. Race/ethnicity was classified as non-Hispanic White, non-Hispanic Black, Hispanic, non-Hispanic Asian, non-Hispanic American Indian/Alaska Native, non-Hispanic Native Hawaiian/Pacific Islander, or other/multiple races. Urbanicity status was derived based on the centroid of the postal code of residence, categorized as metropolitan statistical area (MSA) principal city, MSA nonprincipal city, or non-MSA. Social vulnerability index (SVI) was categorized as low, moderate, or high based on county of residence (CDC/Agency for Toxic Substances and Disease Registry) by using tertiles of SVI score (*7*).

We analyzed data by using SAS version 9.4 (https://www.sas.com) and SUDAAN version 11.0.1 (https://www.rti.org). We weighted all percentages to represent the noninstitutionalized US adult population and calibrated survey weights by age and sex to state-level vaccine administration data reported to CDC as of the middle of the monthly data collection period (*6*). We conducted multivariable logistic regression analysis and predictive marginals to assess factors associated with receipt of a booster dose among adults and generated the unadjusted prevalence ratio (PR) and the adjusted prevalence ratio (aPR) from regression models. We used PR to assess association instead of odds ratio [OR] in our analysis because PR is a more direct measure of effect than OR, and when outcomes are not rare, as with most of vaccination coverage analysis, the OR tends to present an exaggerated measure of effect compared with the PR. We used t-tests to determine differences between groups with statistical significance at p<0.05 and for linear trends over months. This activity was reviewed by CDC and was conducted consistent with applicable federal law and CDC policy (45 C.F.R. part 46.102(l)(2), 21 C.F.R. part 56; 42 U.S.C. §241(d); 5 U.S.C. §552a; 44 U.S.C. §3501).

## Results

COVID-19 booster dose coverage among fully vaccinated adults >18 years of age increased from 25.7% in November 2021 to 63.4% in March 2022 (p<0.05 by test for trend) (Figure). Coverage in mid-March 2022 among those 50–64 years of age (66.4%) and >65 years of age (79.5%) was higher than among those 18–49 years of age (53.6%) (Table 1). By mid-March 2022, booster dose coverage was 52.8% among all adults >18 years of age (including unvaccinated adults in the denominator), and coverage among those 50–64 years of age (58.5%) and >65 years of age (77.0%) was higher than among those 18–49 years of age (40.6%).

**Figure:**
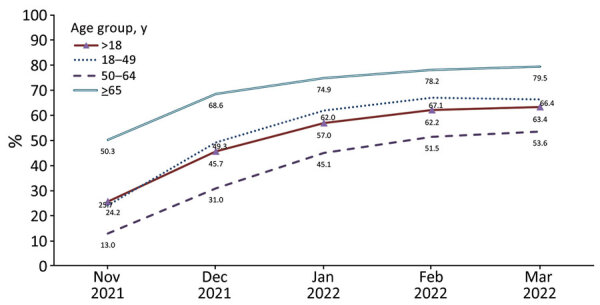
Trends in COVID-19 booster dose vaccination coverage among fully vaccinated adults, by age group, National Immunization Survey–Adult COVID Module, United States, November 2021–March 2022.

**Table 1:** COVID-19 booster dose vaccination coverage for fully vaccinated adults (all ages) by demographic, confidence, or behavior characteristics, National Immunization Survey-Adult COVID Module, United States, February 27–March 26, 2022*

Characteristic	No.	COVID-19 booster dose vaccination coverage, % (95% CI)			
		Persons >18 y of age, n = 63,072	Persons 18–49 y of age, n = 29,084	Persons 50–64 y of age, n = 17,414	Persons >65 y of age, n = 15,527
Total	63,072	63.4 (62.5‒64.3)	53.6 (52.3‒54.9)	66.4 (64.7‒68.1)†	79.5 (78.0‒80.9)†
Sex					
M‡	30,699	61.6 (60.3‒62.9)	50.2 (48.3‒52.0)	66.2 (63.6‒68.6)†	81.2 (79.1‒83.1)†
F	31,853	64.9 (63.7‒66.1)§	56.8 (55.0‒58.6)§	66.7 (64.3‒68.9†	78.2 (76.0‒80.2)†§
Race/ethnicity					
Non-Hispanic White‡	41,878	67.7 (66.7‒68.8)	57.6 (56.0‒59.2)	70.0 (68.0‒71.8)†	80.2 (78.4‒81.8)†
Non-Hispanic Black	6,080	52.7 (50.2‒55.2)§	40.1 (36.6‒43.7)§	56.2 (51.6‒60.7)†§	73.2 (68.7‒77.3)†§
Hispanic	7,423	55.5 (52.8‒58.1)§	47.5 (44.2‒50.9)§	63.1 (57.3‒68.5)†§	82.6 (76.5‒87.3)§
Non-Hispanic Asian	2,754	74.6 (70.8‒78.1)§	72.8 (68.6‒76.7)§	77.9 (64.3‒87.4)	89.6 (81.8‒94.3)†§
Non-Hispanic AI/AN	723	56.6 (47.8‒64.9)§	45.2 (32.6‒58.4)	64.4 (50.9‒75.9)§	76.2 (60.7‒86.9)§
Native Hawaiian/Pacific Islander	296	45.4 (30.2‒61.5)§	40.5 (24.2‒59.2)	38.6 (15.3‒68.7)§	83.3 (54.3‒95.5)†
Non-Hispanic other/multiple races	2,076	54.1 (48.3‒59.9)§	46.3 (39.1‒53.7)§	53.2 (40.7‒65.4)§	83.2 (76.0‒88.6)†
Urbanicity					
MSA, principal city‡	22,652	6.5 (62.0‒65.0)	56.2 (54.1‒58.2)	66.5 (63.2‒ 69.7)†	79.5 (76.5‒82.2)†
MSA, nonprincipal city	28,757	63.9 (62.7‒65.2)	52.9 (51.0‒54.7)§	68.0 (65.7‒70.2)†	80.4 (78.3‒82.3)†
Non-MSA	11,663	60.8 (58.5‒63.0)§	46.6 (43.0‒50.2)§	60.0 (55.7, 64.2)†§	76.7 (73.1‒79.9)†
SVI of county of residence¶					
Low‡	19,979	68.2 (66.8‒69.6)	59.8 (57.6‒61.9)	70.8 (68.3‒73.3)†	81.8 (79.3, 84.1)†
Moderate	21,573	64.8 (63.3‒ 66.2)§	54.8 (52.7‒56.9)§	68.4 (65.5‒71.3)†	80.6 (78.1, 83.0)†
High	15,158	59.3 (57.5‒61.2)§	49.1 (46.4‒51.7)§	62.0 (58.4‒65.5)†§	78.2 (75.1, 81.1)†
Household income					
Below poverty‡	5,216	51.6 (48.2‒54.9)	43.6 (39.1‒48.2)	51.9 (45.3‒58.4)†	69.5 (62.7‒75.6)†
Above poverty, <$75k	19,645	59.8 (58.2‒61.4)§	46.9 (44.6‒49.2)	63.3 (59.9‒66.5)†§	79.9 (77.6‒82.1)†§
Above poverty, >$75k	24,821	70.4 (69.1‒71.7)§	62.9 (60.9‒64.8)§	73.5 (71.1‒75.7)†§	85.3 (82.8‒87.4)†§
Unknown	13,390	61.5 (59.6‒63.5)§	51.5 (48.4‒54.5)§	61.6 (57.5‒65.5)†§	75.8 (72.5‒78.9)†
Education level					
High school graduate or less‡	14,975	53.4 (51.6‒55.1)	39.7 (37.2‒42.3)	56.4 (53.2‒59.6)†	73.9 (71.0‒76.6)†
Some college	17,487	61.3 (59.6‒62.9)§	49.8 (47.5‒52.2)§	66.4 (63.2‒69.5)†§	78.9 (76.0‒81.5)†§
College graduate	28,929	75.7 (74.6‒76.8)§	69.7 (68.0‒71.3)§	78.3 (76.0‒80.4)†§	87.2 (85.4‒88.9)†§
Health insurance					
Insured	57,161	65.3 (64.4‒66.2)§	55.8 (54.4‒57.1)§	67.8 (66.0‒69.5)†§	79.9 (78.4‒81.3)†§
Not insured‡	4,229	41.2 (37.6‒44.9)	37.2 (33.0‒41.6)	51.7 (44.3‒59.0)†	58.1 (39.5‒74.6)†
Foreign born status					
Foreign born	7,707	63.1 (60.6‒65.6)	55.8 (52.5‒59.1)	68.5 (63.2‒73.4)†	81.9 (76.7‒86.0)†
US born‡	52,546	63.9 (62.9‒64.9)	53.7 (52.2‒55.1)	66.5 (64.6‒68.3)†	79.9 (78.3‒81.4)†
Frontline and essential workers#					
Essential healthcare	6,580	67.4 (64.8‒69.9)§	62.5 (59.2‒65.6)§	72.6 (67.5‒77.1)†§	85.4 (77.3‒91.0)†§
School and childcare	2,321	68.2 (63.3‒72.8)§	68.3 (62.1‒73.9§	66.2 (56.7‒74.5)	83.8 (73.4‒90.6)†
Other frontline worker	3,870	52.2 (48.5‒55.9)	45.1 (40.4‒49.9)	64.5 (58.1‒70.3)†	75.6 (64.9‒83.8)†
Other essential worker‡	6,228	52.0 (49.1‒54.9)	42.9 (39.3‒46.7)	64.2 (59.0‒69.1)†	73.4 (64.1‒81.0)†
Persons not essential workers	43,658	65.4 (64.3‒66.4)§	54.3 (52.6‒55.9)§	66.2 (64.1‒68.3)†	79.6 (78.0‒81.1)†
Disability**					
Yes (any)	6,122	62.2 (59.4‒64.9)	43.8 (38.2‒49.5)§	56.8 (51.5‒61.9)†§	76.1 (72.5‒79.4)†§
No‡	56,807	63.5 (62.6‒64.5)	54.2 (52.8‒55.5)	67.6 (65.8‒69.4)†	80.3 (78.7‒81.9)†
Ever had COVID-19					
Yes	21,202	50.9 (49.3‒52.5)§	44.8 (42.7‒46.8)§	53.2 (50.2‒56.3)†§	69.8 (65.9‒73.5)†§
No‡	41,058	69.5 (68.4‒70.5)	59.4 (57.7‒61.1)	72.4 (70.4‒74.4)†	82.1 (80.5‒83.6)†
Received any vaccine that was not a COVID-19 vaccine in the past 2 y					
Yes	37,874	73.6 (72.6‒74.6)§	65.5 (63.8‒67.1)§	75.8 (73.8‒77.7)†§	83.0 (81.3‒84.6)†§
No‡	24,836	46.4 (44.9‒48.0)	39.5 (37.5‒41.5)	49.2 (46.2‒52.2)†	68.4 (64.9‒71.8)†
Reported medical conditions					
Yes	19,165	69.9 (68.4‒71.3)§	59.4 (56.5‒62.3)§	67.9 (65.1‒70.5)†	80.9 (78.8‒82.8)†
No‡	43,274	60.4 (59.3‒61.5)	52.2 (50.7‒53.7)	65.4 (63.2‒67.6)†	78.7 (76.5‒80.7)†
Concerned about getting COVID-19					
Not at all/a little‡	37,362	58.1 (56.9‒59.3)	49.1 (47.4‒50.7)	61.8 (59.4‒64.1)†	75.7 (73.5‒77.8)†
Moderately/very	25,456	70.1 (68.8‒71.4)§	60.5 (58.4‒62.6)§	71.5 (68.9‒74.0)†§	83.5 (81.4‒85.3)†§
Thinks COVID-19 vaccine is important					
Not at all/a little‡	10,815	22.8 (20.4‒25.3)	16.9 (14.5‒19.7)	26.0 (21.2‒31.4)†	41.7 (33.9‒49.9)†
Very/completely	51,384	67.8 (66.9‒68.7)§	58.9 (57.5‒60.2)§	70.2 (68.5‒71.9)†§	82.0 (80.5‒83.4)†§
Thinks COVID-19 vaccine is safe					
Not at all/somewhat‡	17,737	39.8 (37.9‒41.7)	30.5 (28.1‒33.0)	42.1 (38.6‒45.5)§	62.5 (58.3‒66.6)†
Very/completely	42,063	71.8 (70.8‒72.7)§	62.9 (61.4‒64.3)§	75.4 (73.4‒77.2)†§	84.3 (82.7‒85.7)†§
Friends and family vaccinated					
No/some family or friends‡	12,740	42.5 (40.2‒44.8)	31.2 (28.1‒34.5)	47.0 (42.5‒51.6)†	60.9 (56.1‒65.5)†
Many/almost all family or friends	49,514	67.0 (66.0‒67.9)§	57.5 (56.1‒58.9)§	69.9 (68.0‒71.7)†§	82.6 (81.1‒84.0)†§
Difficulty getting a COVID-19 vaccine					
Very/somewhat‡	7,848	73.0 (70.7‒75.1)	62.5 (58.7‒66.2)	72.6 (68.3‒76.6)†	88.0 (85.1‒90.4)†
A little/not at all	54,901	61.9 (60.9‒62.9)§	52.4 (51.0‒53.8)§	65.5 (63.6‒67.3)†§	77.8 (76.1‒79.4)†§

*Values are percentages (95% CIs). Percentages are weighted. AI/AN, non-Hispanic American Indian/Alaska Native; MSA, metropolitan statistical area; SVI, Social Vulnerability Index. †p<0.05 by t-test for comparisons of vaccination coverage within age groups using adults 18–49 y as the reference level. ‡Reference level for comparisons of vaccination coverage within each variable. §p<0.05 by t-test for comparisons of vaccination coverage within each variable with the indicated reference level. ¶Centers for Disease Control and Prevention/Agency for Toxic Substances and Disease Registry SVI uses 15 US census variables to help officials identify communities that might need support before, during, or after disasters. #Essential worker groups were categorized as essential healthcare personnel (including healthcare, social service, and death care workers), school and childcare (including preschool or daycare, K–12 school, and other schools and instructional settings), other frontline (including first response [e.g., police or fire protection], correctional facility, food and beverage store, agriculture, forestry, fishing, or hunting, food manufacturing facility, nonfood manufacturing facility, public transit, and US Postal Service), other essential (including other essential that are not listed above), and not a frontline or essential worker (including those who were not employed). **Defined as an affirmative response to the following survey question: “Do you have serious difficulty seeing, hearing, walking, remembering, making decisions, or communicating?”

Among fully vaccinated adults >18 years of age, booster dose coverage in mid-March 2022 was lower among Native Hawaiian/Pacific Islander (45.4%), Black (52.7%), Other/multiple races (54.1%), Hispanic (55.5%), and American Indian/Alaska Native (56.6%) than among White adults (67.7%); Asian adults had the highest coverage (74.6%) (p<0.05) (Table 1). Booster dose coverage was higher among all healthcare personnel (HCP) >18 years of age and among school and childcare workers 18–49 years of age than for other essential workers (Table 1).

Coverage was higher for adults who had reported medical conditions (69.9%) than in adults who did not have these conditions (60.4%). In addition, women and those who lived above the poverty level, had some college or higher education, had health insurance, and had received a vaccine other than COVID-19 in the past 2 years had higher booster vaccination coverage than did the respective reference groups (Table 1). Adults living in a moderate or high SVI county and those who had a previous COVID-19 infection had lower booster dose vaccination coverage than did the respective reference groups. Adults with disability had lower booster dose vaccination coverage than did adults without disability across age groups (18–49, 50–64, and >65 years of age) (Table 1). Furthermore, compared with the respective reference groups, booster dose coverage was higher among adults who reported they were concerned about getting COVID-19 (70.1% vs. 58.1%), thought the vaccine was safe (71.8% vs. 39.8%), and thought the vaccine was useful for protection from COVID-19 (67.8% vs. 22.8%). In addition, reporting little or no difficulty getting a COVID-19 vaccine was associated with decreased booster vaccination (Table 1).

In the multivariable model adjusted for demographic variables, characteristics independently associated with increased booster vaccination were older age, Asian race, household income >$75,000, some college or higher education, being insured, having received any vaccine that was not a COVID-19 vaccine in the past 2 years, and having reported medical conditions (Table 2). In addition, for occupational categories, being an HCP, school/childcare worker, or other frontline worker, or not being an essential worker, was associated with increased booster vaccination compared with being in the category of other essential worker. Non-Hispanic Black adults, those living in a high SVI county, those living in non-MSAs, those with a disability, and those with a previous COVID-19 infection had decreased booster vaccination. For the multivariable model including demographic and behavioral variables, demographic characteristics independently associated with booster vaccination were similar to those for the model adjusted for demographic variables only. In addition, being concerned about getting COVID-19, believing the vaccine is safe, believing the vaccine is useful for protection, and having many or almost all friends and family vaccinated were independently associated with increased booster vaccination. Reporting a little or no difficulty getting a COVID-19 vaccine was independently associated with decreased booster vaccination (Table 2).

**Table 2:** Logistic model of associations between COVID-19 booster dose vaccination and characteristics among fully vaccinated adults, National Immunization Survey-Adult COVID Module, United States, February 27‒March 26, 2022*

Characteristic	PR (95% CI)	aPR (95% CI) demographic variables	aPR (95% CI) demographic and behavior variables
Age group, y			
18–49	Referent	Referent	Referent
50–64	1.24 (1.20‒1.28)†	1.18 (1.14‒1.22)†	1.15 (1.11‒1.19)†
>65	1.48 (1.44‒1.53)†	1.38 (1.33‒1.42)†	1.32 (1.28‒1.36)†
Sex			
M	Referent	Referent	Referent
F	1.05 (1.02‒1.08)†	1.00 (0.98‒1.03)	0.99 (0.97‒1.02)
Race/ethnicity			
Non-Hispanic White	Referent	Referent	Referent
Non-Hispanic Black	0.78 (0.74‒0.82)†	0.89 (0.85‒0.93)†	0.88 (0.84‒0.92)†
Hispanic	0.82 (0.78‒0.86)†	1.00 (0.96‒1.05)	0.99 (0.94‒1.03)
Non-Hispanic Asian	1.10 (1.05‒1.16)†	1.14 (1.08‒1.1)†	1.12 (1.06‒1.19)†
Non-Hispanic AI/AN	0.84 (0.72‒0.97)†	0.98 (0.87‒1.10)	0.99 (0.88‒1.12)
Native Hawaiian/Pacific Islander	0.67 (0.47‒0.96)†	1.03 (0.87‒1.21)	1.08 (0.89‒1.30)
Non-Hispanic other/multiple races	0.80 (0.72‒0.89)†	0.94 (0.85‒1.03)	0.95 (0.87‒1.04)
Urbanicity			
MSA, principal city	Referent	Referent	Referent
MSA, nonprincipal city	1.01 (0.98‒1.04)	0.97 (0.94‒1.00)	0.98 (0.95‒1.01)
Non-MSA	0.96 (0.92‒1.00)	0.91 (0.87‒0.95)†	0.95 (0.91‒0.99)†
SVI of county of residence‡			
Low	Referent	Referent	Referent
Moderate	0.95 (0.92‒0.98)†	0.97 (0.95‒1.00)	0.98 (0.96‒1.01)
High	0.87 (0.84‒0.90)†	0.95 (0.92‒0.99)†	0.98 (0.95‒1.01)
Household income			
Below poverty	Referent	Referent	Referent
Above poverty, <$75k	1.16 (1.08‒1.24)†	1.06 (1.00‒1.13)	1.05 (0.99‒1.11)
Above poverty, >$75k	1.37 (1.28‒1.46)†	1.15 (1.08‒1.22)†	1.13 (1.06‒1.20)†
Unknown	1.19 (1.11‒1.28)†	1.09 (1.02‒1.17)†	1.09 (1.03‒1.17)†
Education level			
High school graduate or less	Referent	Referent	Referent
Some college	1.15 (1.10‒1.20)†	1.09 (1.05‒1.13)†	1.06 (1.02‒1.10)†
College graduate	1.42 (1.37‒1.47)†	1.27 (1.22‒1.32 †	1.19 (1.15‒1.23)†
Health insurance			
Insured	1.58 (1.45‒1.73)†	1.11 (1.04‒1.19)†	1.13 (1.06‒1.20)†
Not insured	Referent	Referent	Referent
Foreign born status			
Foreign born	0.99 (0.95‒1.03)	1.04 (0.99‒1.09)	1.02 (0.98‒1.07)
US born	Referent	Referent	Referent
Frontline and essential workers§			
Essential healthcare	1.30 (1.21‒1.39)†	1.13 (1.07‒1.20)†	1.11 (1.05‒1.17)†
School and childcare	1.31 (1.20‒1.43)†	1.13 (1.04‒1.22)†	1.10 (1.02‒1.18)†
Other frontline worker	1.00 (0.92‒1.10)	1.08 (1.01‒1.15)†	1.06 (1.00‒1.13)
Other essential worker	Referent	Referent	Referent
Persons who are not essential workers	1.26 (1.19‒1.33)†	1.07 (1.02‒1.12)†	1.04 (0.99‒1.08)
Disability¶			
Yes (any)	0.98 (0.93‒1.02)	0.93 (0.88‒0.97)†	0.94 (0.90‒0.99)†
No	Referent	Referent	Referent
Ever had COVID-19			
Yes	0.73 (0.71‒0.76)†	0.81 (0.78‒0.83)†	0.85 (0.82‒0.87)†
No	Referent	Referent	Referent
Received any vaccine that was not a COVID-19 vaccine in the past 2 y			
Yes	1.59 (1.53‒1.64)†	1.35 (1.31‒1.40)†	1.27 (1.23‒1.31)†
No	Referent	Referent	Referent
Reported medical conditions			
Yes	1.16 (1.13‒1.19)†	1.06 (1.02‒1.09)†	1.02 (0.99‒1.05)
No	Referent	Referent	Referent
Concerned about getting COVID-19			
Not at all/a little	Referent	NA	Referent
Moderately/very	1.21 (1.17‒1.24)†		1.06 (1.03‒1.09)†
Thinks COVID-19 vaccine is important			
Not at all/a little	Referent	NA	Referent
Very/completely	2.98 (2.67‒3.31)†		1.54 (1.42‒1.69)†
Thinks COVID-19 vaccine is safe			
Not at all/somewhat	Referent	NA	Referent
Very/completely	1.80 (1.72‒1.89)†		1.30 (1.25‒1.36)†
Friends and family vaccinated			
No/some family or friends	Referent	NA	Referent
Many/almost all family or friends	1.58 (1.49‒1.67)†		1.14 (1.09‒1.19)†
Difficulty getting a COVID-19 vaccine			
Very/somewhat	Referent	NA	Referent
A little/not at all	0.85 (0.82‒0.88)†		0.93 (0.90‒0.96)†

*AI/AN, non-Hispanic American Indian/Alaska Native; aPR, adjusted prevalence ratio; MSA, metropolitan statistical area; NA, not applicable; PR, prevalence ratio; SVI, Social Vulnerability Index. †p<0.05 by t-test for comparison within each covariate category with the indicated reference level. ‡The Centers for Disease Control and Prevention/Agency for Toxic Substances and Disease Registry SVI uses 15 US census variables to help officials identify communities that might need support before, during, or after disasters. §Essential worker groups were categorized as essential healthcare personnel (including healthcare, social service, and death care workers), school and childcare (including preschool or daycare, K–12 school, and other schools and instructional settings), other frontline (including first response [e.g., police or fire protection], correctional facility, food and beverage store, agriculture, forestry, fishing, or hunting, food manufacturing facility, nonfood manufacturing facility, public transit, and US Postal Service), other essential (including other essential that are not listed above), and not a frontline or essential worker (including those who were not employed). ¶Defined as an affirmative response to the following survey question: “Do you have serious difficulty seeing, hearing, walking, remembering, making decisions, or communicating?”

Among adults >18 years of age who were fully vaccinated but did not receive a booster dose, prevalence of provider recommendation of COVID-19 vaccine was 47.9%, and prevalence was higher among adults 50–64 years of age (50.7%) than among adults 18–49 years (46.5%). Overall, 36.8% reported “being concerned about getting COVID-19,” 60.6% reported “thinking the vaccine is safe,” 80.5% reported “believing COVID-19 vaccine is important for protection from infection,” 77.6% reported “most or almost all friends or family were vaccinated,” and 31.9% reported “work or school requires COVID-19 vaccine” (Table 3). Among fully vaccinated adults who did not receive a booster dose, ≈4%–10% of adults reported difficulties in getting a COVID-19 vaccine (e.g., difficulty getting vaccinated [9.9%], difficulty getting an appointment online [9.5%], difficulty knowing where to get vaccinated [5.3%], and difficulty getting to vaccination sites [4.2%]) (Table 3).

**Table 3:** Characteristics of persons who were fully vaccinated but did not receive booster dose vaccination, National Immunization Survey-Adult COVID Module, February 27‒March 26, 2022*

Characteristic	Persons >18 y of age, n = 16,790	Persons 18–49 y of age, n = 9,378	Persons 50–64 y of age, n = 4,394	Persons >65 y of age, n = 2,767
Confidence, behavior, experience, provider recommendation, and requirement for adults fully vaccinated but who did not receive booster dose†				
Concerned about getting COVID-19, strongly or moderately	36.8 (35.3‒38.4)	34.1 (32.2‒36.1)	41.6 (38.4‒44.9)‡	41.1 (37.2‒45.2)‡
Thinks COVID-19 vaccine is safe, completely or mostly	60.6 (59.0‒62.1)	60.5 (58.4‒62.5)	59.5 (56.2‒62.6)	64.4 (60.4‒68.3)
Thinks COVID-19 vaccine is needed for protection, mostly or somewhat	80.5 (79.2‒81.8)	78.9 (77.1‒80.6)	82.5 (80.1‒84.8)‡	84.4 (81.3‒87.1)‡
Had friends/family who were vaccinated, almost all or many	77.6 (76.3‒78.9)	78.6 (76.9‒80.2)	76.6 (73.8‒79.2)	74.4 (70.6‒77.8)‡
Work or school requires COVID-19 vaccine	31.9 (30.4‒33.4)	39.7 (37.7‒41.7)	24.5 (21.8‒27.4)‡	8.3 (6.3‒10.8)‡
Provider recommendation of the COVID-19 vaccine	47.9 (46.3‒49.4)	46.5 (44.5‒48.5)	50.7 (47.4‒53.9)‡	49.9 (45.9‒54.0)
Difficulty for adults fully vaccinated but did not receive booster dose§				
Getting vaccinated, mostly or somewhat	9.9 (9.1‒10.9)	9.5 (8.3‒10.7)	11.2 (9.4‒13.3)	9.6 (7.7‒11.9)
Getting an appointment online	9.5 (8.6‒10.5)	8.7 (7.6‒9.8)	10.5 (8.7‒12.7)	11.1 (8.6‒14.2)
Not knowing where to get vaccinated	5.3 (4.7‒6.1)	5.3 (4.4‒6.3)	5.5 (4.3‒7.0)	4.9 (3.7‒6.5)
Getting to vaccination sites	4.2 (3.6‒4.9)	4.3 (3.5‒5.2)	4.0 (3.0‒5.2)	3.9 (2.6‒5.7)
Vaccination sites are not open at convenient times	5.1 (4.5‒ 5.7)	5.6 (4.8‒6.7)	4.4 (3.5‒5.5)	3.5 (2.6‒4.6)‡

*Values are percentages (95% CIs). Percentages are weighted. †Questions were asked about COVID-19 vaccination generally and not specifically about COVID-19 booster dose vaccination. ‡p<0.05 by t-test for comparisons with adults 18–49 y of age as the reference level. §Questions were asked about difficulty getting a COVID-19 vaccination and did not ask specifically about difficulty getting a booster vaccine.

## Discussion

By March 2022, a total of 84% of American adults were fully vaccinated with the COVID-19 primary vaccine series, according to the NIS-ACM (*7*). Booster dose coverage among fully vaccinated adults >18 years of age was 63.4% in March 2022. Overall, ≈53% of the adult population have both received the primary series and >1 booster vaccination. Disparities by race/ethnicity and other factors are apparent in booster dose uptake. Healthcare providers can educate and encourage everyone to receive a booster dose when they are eligible. Targeted strategies are needed to reduce disparities in COVID-19 vaccination coverage toward reducing disparities in COVID-19.

Booster dose vaccine uptake was most strongly associated with confidence in the need for getting vaccinated, confidence in vaccine safety, and concern about getting COVID-19. Although persons were presumably amenable to getting the primary vaccine series, ≈39% of fully vaccinated adults who did not receive a booster dose did not believe COVID-19 vaccines were completely safe, and 20% did not believe they were useful for protection against COVID-19. Most fully vaccinated adults who did not receive a booster (63%) were not concerned about getting COVID-19, especially younger adults. Higher levels of concern about COVID-19 and positive attitudes toward vaccination among adults might contribute to uptake of booster dose vaccination. To further improve vaccine uptake, more innovative approaches are needed to improve vaccine confidence.

In addition, we found that reporting of family and friends being vaccinated was associated with booster dose vaccination uptake. This finding indicated the useful role of social processes for increasing vaccination (*8**,**9*). Community healthcare workers can educate the community about the vaccines, text persons to let them know of vaccine eligibility, use public media or social media, and encourage vaccinated community members to share their own vaccination experiences with their unvaccinated friends and family as a means for improving COVID-19 vaccination coverage (*8**–**10*).

Our analysis did not find an association between increasing levels of difficulty accessing vaccine and lower booster dose vaccination coverage. This finding might be attributable to extensive efforts to reduce access barriers, including mobile vaccination sites, removing an insurance or identification requirement, and substantial community-led outreach (*11**–**13*). We found that persons who reported difficulty getting a COVID vaccine had higher booster dose coverage, a finding that might seem counterintuitive. However, our study assessed barriers to COVID-19 vaccination overall and not specifically for booster vaccines. Early adopters who sought vaccine at the beginning of the COVID-19 vaccination program when supply was scarce might have been more likely to experience barriers to vaccination. The finding that factors such as lower income and education were associated with lower booster uptake, even after controlling for attitudinal factors, suggests that barriers to access might remain. Among fully vaccinated adults who did not receive booster dose vaccination, ≈4%–10% of adults did report difficulties in getting a COVID-19 vaccine (e.g., difficulty getting vaccinated, getting an appointment online, knowing where to get vaccinated, or getting to vaccination sites). Understanding the barriers to vaccination can help identify strategies most likely to increase vaccine uptake. Many of these barriers could be further reduced by providing vaccination in the office of their usual medical provider (*14*,*15*). Reducing barriers to COVID-19 vaccination could further improve vaccination coverage among adults.

COVID-19 has disproportionately impacted racial and ethnic minority populations by illness, hospitalizations, and death in the United States (*16*,*17*). Although most disparities in primary COVID-19 vaccination had been eliminated by March 2022 (*7*), disparities in booster dose vaccination remain. Booster coverage was lower among all racial/ethnic groups except Asian compared with non-Hispanic White adults. Equitable vaccination can help to reduce illness-related disparities in minority groups. One study found that COVID-19 vaccine hesitancy decreased more rapidly among non-Hispanic Black than non-Hispanic White adults during December 2020‒June 2021, indicating that lower coverage might be less likely the result of vaccine hesitancy than other factors (*18*). Several factors, including knowledge, attitudes, and beliefs about vaccines and barriers related to accessing vaccines and healthcare services, contribute to lower vaccination coverage in non-Hispanic Black adults and other minority groups (*18**–**21*). Tailored and community-led interventions, including postal code‒level vaccination access planning and community engagement, have been shown to reduce inequities in COVID-19 vaccination by race and ethnicity (*10*,*22*,*23*). Vaccination programs could implement culturally and linguistically appropriate focused interventions among communities with lower vaccination coverage to reduce vaccination disparities.

COVID-19 booster dose vaccination coverage was particularly lower among adults living in poverty, with lower education, or without health insurance, and continued efforts are needed to reach these groups and reduce inequities (*24**–**26*). In addition, having a previous COVID-19 infection was independently associated with decreased booster vaccination. We did not assess when persons had COVID-19 in relation to the timing of the initial vaccination series or booster vaccination, but this finding might suggest that persons who have already had COVID-19 might believe that they are protected and do not need a booster. However, COVID-19 vaccines including booster doses have been shown to provide additional protection to persons who had previous infections (*27*), and all adults are recommended to receive a booster dose, regardless of previous infection with COVID-19.

Although we were unable to assess provider recommendation specifically for booster vaccination, studies have shown that a provider recommendation is highly associated with vaccine uptake (*8**,**25**,**28*). Findings from our study indicated that, among those who have not received a booster dose, >50% have not received a provider recommendation for any COVID-19 vaccine. Underuse of primary care services during the COVID-19 pandemic and some adults not having a primary physician for sick or preventive care (*29*) might have limited opportunities for providers to convey recommendations and communicate with patients the benefits of primary and booster dose vaccination and information on the safety and effectiveness of COVID-19 vaccination. Clinicians and healthcare providers, including pharmacists and allied health professionals, can get coaching, practice, and support from the broader healthcare organizations in which they are embedded, follow the Advisory Committee on Immunization Practices (ACIP) recommendations (*1**,**2*), recommend needed vaccinations, and encourage eligible persons to receive COVID-19 booster dose vaccination.

Findings from this study showed that booster dose vaccination coverage among adults 50–64 and >65 years of age was higher than among those 18–49 years of age, and this pattern remained the same after controlling for demographic and behavioral variables. The risk for severe illness from COVID-19 increases with age (*30*,*31*). Higher COVID-19 booster dose vaccination coverage among older adults might also have been caused by early recommendation from ACIP for this population (*1*,*2*). Booster dose coverage was higher among persons who had reported medical conditions but lower among persons who had disabilities. Higher COVID-19 booster dose vaccination coverage among adults who had reported medical conditions might also be caused by the early recommendation in September 2021 from ACIP and recognition of increased risk for severe COVID-19 in this population (*1*,*2*). One recent study indicated that adults who had disabilities experienced more difficulty in obtaining a COVID-19 vaccination than persons who did not have a disability (*26*). Healthcare providers can try to ensure that all persons receive COVID-19 vaccination if they are eligible, regardless of age. Reducing barriers to scheduling and making vaccination sites more accessible might help improve vaccination coverage among adults who have disabilities (*26*).

Results from our study showed that booster dose coverage was much higher among essential HCP than among other essential workers. Although primary series vaccination was >90% among HCP and those in the education sector and ≈80% for other frontline and essential workers (*7*), booster dose coverage was much lower, leaving populations that have frequent COVID-19 exposure possibly susceptible to disease. This finding could be caused by lack of requirements for vaccination and removal of programs such as onsite vaccination that were available for the initial vaccine series. In addition, other access programs for different populations were probably available for initial vaccines but not for boosters. Reinstating these more intensive access programs or putting vaccines in the hands of primary care providers could help increase booster dose coverage among this population.

Four limitations might be considered when interpreting these findings. First, NIS-ACM has a low response rate (≈22%). However, survey weights were calibrated to COVID-19 vaccine administration data to mitigate possible bias from incomplete sample frame, nonresponse, and misclassification of vaccination status. Second, COVID-19 vaccination was self-reported and might be subject to recall or social desirability bias. However, reliability of self-reported COVID-19 vaccination might be comparable with that of self-report of influenza vaccination, which has been shown to have a relatively high agreement with vaccination status ascertained from medical records (*32**,**33*). Third, provider recommendations or work/school requirements for COVID-19 vaccination, and vaccination access barriers were not specifically assessed for booster doses.

Booster dose coverage was not optimal, and disparities by race/ethnicity and other factors are apparent in booster dose uptake. Continual monitoring of booster dose vaccination will be helpful for developing tailored strategies to improve vaccination coverage, especially with the recent recommendation for adults to receive the updated COVID-19 boosters (*34*). The updated COVID-19 boosters are formulated to better protect against the most recently circulating COVID-19 variant. The immune response of the updated COVID-19 booster vaccine was superior to that of the previous booster vaccine, and the safety was similar as that of the previous booster vaccine (*35*). To maximize protection against COVID-19, both an increase in persons initiating and completing the primary series, and getting recommended boosters, is needed.

Targeted strategies are needed to improve booster dose vaccination coverage among adults. These strategies should include healthcare providers educating and encouraging everyone to receive a booster dose when they are eligible regardless of age and previous infection with COVID-19 (*8**–**10*) and provisions for consistent access to vaccines, vaccination incentives, onsite vaccination, and reminders (*14**,**15*). More innovative approaches should include improving confidence in vaccines, community healthcare workers encouraging vaccinated community members to share their own vaccination experiences with their unvaccinated friends and family (*14**,**15*), understanding barriers and reducing barriers to vaccination (*14**,**15*), and providing vaccination in the office of their usual medical provider (*14**,**15*). Vaccination programs are needed that implement culturally and linguistically appropriate focused interventions among communities with lower vaccination coverage (*8**–**10*,*14**,**15*,*26*,*36*) and encourage national, state, and local health departments, and community-based and faith-based organizations to implement a combination of strategies, which have been shown to be effective in improving booster dose coverage (*26*,*36*).
